# Association of oxidative balance score with all-cause and cardiovascular mortality in overweight and obese

**DOI:** 10.3389/fnut.2025.1536024

**Published:** 2025-01-28

**Authors:** Shuxin Ying, Hongyan Ding, Yanjin Chen, Su Zheng

**Affiliations:** Department of Nutrition, Hangzhou Third Hospital Affiliated to Zhejiang Chinese Medical University, Hangzhou, China

**Keywords:** oxidative balance score, mortality, overweight, obesity, cardiovascular mortality

## Abstract

**Background:**

The oxidative balance score (OBS) combines diverse dietary components with lifestyle factors to comprehensively evaluate oxidative stress. The investigation focuses on the link between the OBS and mortality outcomes, including cardiovascular and all-cause deaths, in overweight and obese individuals.

**Methods:**

The analysis utilized data from the National Health and Nutrition Examination Survey (NHANES), covering the period from 1999 to 2018. Mortality information, categorized into all-cause and cardiovascular deaths, was gathered from the National Death Index (NDI). Kaplan–Meier survival analysis, along with multivariate Cox regression and restricted cubic spline (RCS) modeling, were utilized to explore the link between OBS and mortality risks. Subgroup analysis and sensitivity analysis were used to assess the robustness of the results and possible effect modifiers. Mediation analysis identifies pathways through which the independent variable affects the dependent variable.

**Results:**

In this study, 26,219 participants with overweight or obesity were enrolled, with an average age of 49.8 ± 17.4 years. During a median follow-up duration of 115 months, 2,239 participants (8.5%) died, including 837 (3.2%) from cardiovascular disease. According to Kaplan–Meier analysis, mortality was highest among participants in the lowest OBS quartile (Q1) and lowest among those in the highest quartile (Q4). Participants in the fourth OBS quartile experienced a 21.7% decrease in the risk of mortality from all causes and a 29.5% decrease in cardiovascular mortality risk, according to fully adjusted results, compared to those in the first quartile. These results were validated through subgroup analyses. The analysis of RCS revealed a notable inverse association between OBS and mortality outcomes. Mediation analysis indicates that white blood cell count (WBC) and gamma-glutamyl transferase (GGT) serve as significant mediators in the association between OBS and mortality risk.

**Conclusion:**

Elevated levels of OBS were strongly linked to reduced potential for both cardiovascular and all-cause mortality among individuals who are overweight or obese.

## 1 Introduction

The accumulation of excessive or ectopic fat, characteristic of being overweight or obese, greatly raises the likelihood of various metabolic disorders. According to WHO, the percentage of obese adults increased from 7% in 1990 to 16% by 2022. In that year, 43% of adults were categorized as overweight (1, 2). Body Mass Index (BMI) values have also steadily increased, with the average BMI in the United States having reached 27.8 by 2014 (3).

Overweight and obesity are key factors to non-infectious diseases, such as cardiovascular diseases, endocrine diseases, chronic respiratory diseases, and digestive system diseases (4–7). Beyond these health impacts, elevated BMI imposes a significant economic burden. By 2030, global costs associated with overweight and obesity are projected to reach $3 trillion annually, potentially exceeding $18 trillion by 2060 (1). Between 1990 and 2017, high BMI was responsible for a more than twofold rise in deaths and disability-adjusted life years (DALYs) (8).

Oxidative stress, a common pathophysiological condition in obese individuals, is a significant driver of chronic disease development. It arises from a disequilibrium between antioxidant defenses and reactive oxygen species (ROS), where redundant ROS production leads to cellular damage, inflammatory responses, and oxidative injuries, ultimately elevating disease risk (9). The oxidative balance score (OBS) evaluates the balance between the body’s oxidative and antioxidative processes, providing a semi-quantitative measure of oxidative stress. Comprising 15 antioxidants and 5 pro-oxidants, this score is derived from 4 lifestyle components and 16 dietary nutrients, with higher scores suggesting greater antioxidant extent and reduced risk of oxidative stress (10–13).

OBS is increasingly utilized in epidemiological research and has been linked to reduced abdominal obesity (14). Furthermore, levels of OBS have shown a negative correlation with depression (15), non-alcoholic fatty liver disease (NAFLD) (10), cardiovascular disease (16), and diabetes mellitus (17).

As far as we know, earlier research has not delved into the relationship between OBS and mortality risk among overweight or obese individuals. Therefore, the objective of this study is to investigate the link between OBS and mortality from all causes and cardiovascular diseases in overweight and obese, based on data sourced from the National Health and Nutrition Examination Survey (NHANES).

## 2 Methods

### 2.1 Study design and population

The Centers for Disease Control and Prevention (CDC) oversees NHANES, a long-term, nationwide project. It is designed to comprehensively evaluate the physical health and nutritional profiles of individuals in the U.S. and the associated influencing factors. NHANES utilizes an intricate, multistage probabilistic sampling approach to gather data in biennial cycles, comprising five core components: demographic data, dietary information, physical examination data, laboratory test results, and questionnaire. All participants are required to sign written informed consent forms, following detailed scrutiny and authorization from the NCHS Research Ethics Board.

**Figure 1:**
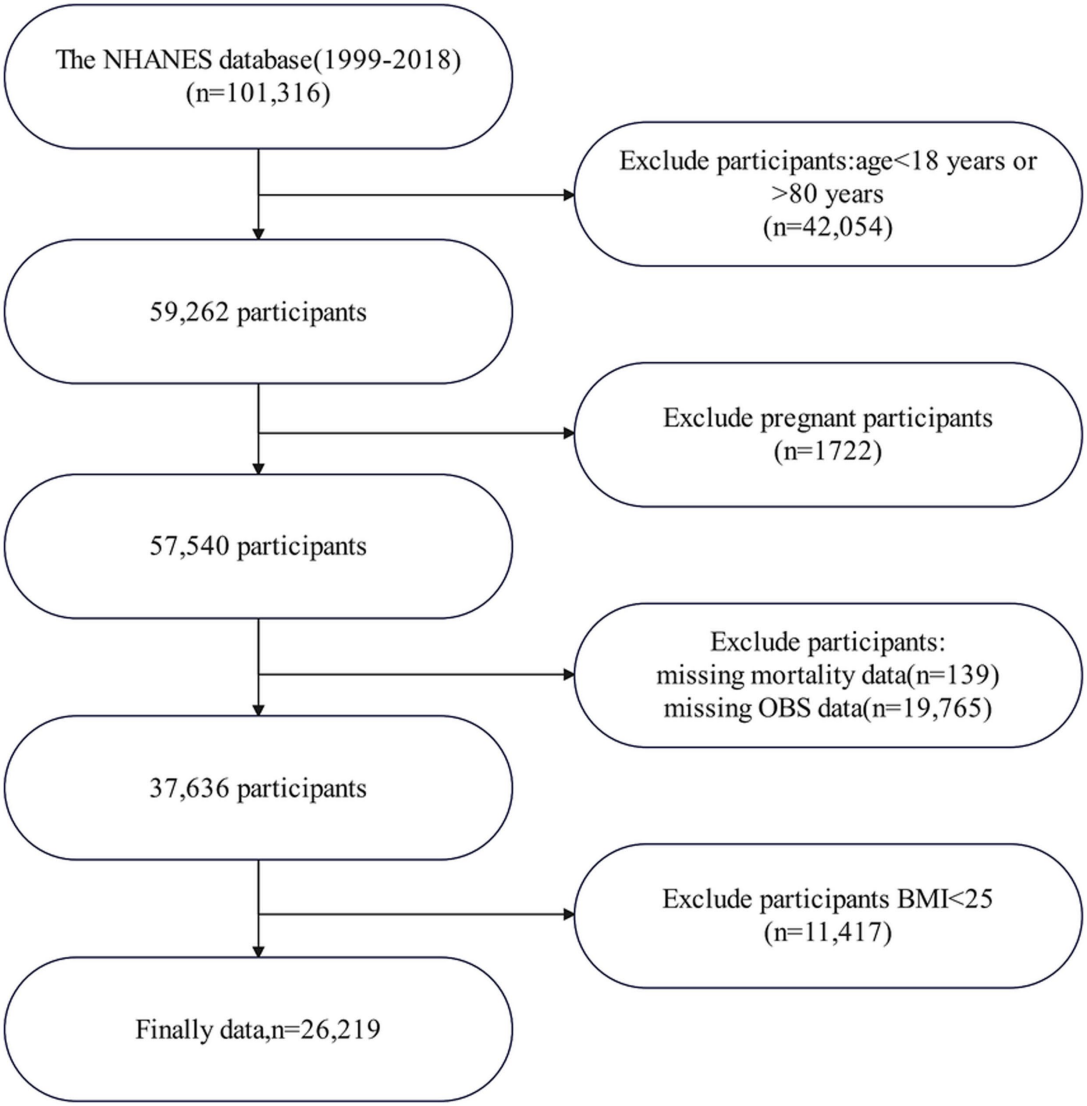
Flowchart of patient enrollment. NHANES, National Health and Nutrition Examination Survey; OBS, oxidative balance score; BMI, body mass index.

This study included overweight and obese adult subjects with complete OBS-related information derived from 10 continuous NHANES cycles conducted between 1999 and 2018 (1999–2000, 2001–2002, 2003–2004, 2005–2006, 2007–2008, 2009–2010, 2011–2012, 2013–2014, 2015–2016, and 2017–2018). The specifics of the inclusion and exclusion criteria are illustrated in Figure 1. Initially, 101,316 participants were recruited; however, 42,054 were excluded for being under 18 or over 80 years of age. Additional exclusions include pregnant women (*n* = 1,722), individuals with incomplete OBS data (*n* = 19,765), unavailable mortality data (*n* = 139), and those with a BMI <25 (*n* = 11,417). Ultimately, 26,219 participants remained for analysis (Figure 1).

### 2.2 Calculation of OBSs

Due to differences in data across years, dietary data from 1999 to 2002 were based on total nutrient intakes, while dietary data from 2003 to 2018 were derived from the first day’s total nutrient intakes. OBS incorporates 16 dietary components, such as niacin, riboflavin, total folate, vitamin E, vitamin B6, vitamin B12, vitamin C, carotenoids, and dietary fiber. It also includes minerals like selenium, calcium, magnesium, zinc, and copper, as well as iron and total fat. In addition to these dietary factors, four lifestyle factors are considered: smoking behavior, alcohol intake, BMI, and physical activity. Out of these, five elements—BMI, total fat, iron, smoking habits, and alcohol consumption—are recognized as pro-oxidants, whereas the other elements are categorized as antioxidants (13). For the antioxidant components, scores are assigned based on tertiles: 0 for the first tertile, 1 for the second, and 2 for the third. On the other hand, for pro-oxidant components, the scoring is reversed, with the first tertile receiving a score of 2 and the third tertile being assigned a score of 0 (18).

The physical activity (PA) levels were determined using the NHANES Physical Activity Questionnaire (PAQ), and the calculation relied on the formula: the metabolic equivalent of task (MET) score * duration * weekly frequency. Due to variations in the PAQ across different cycles, PA calculations were adjusted according to the corresponding survey years. From 1999 to 2006, PA was categorized into walking or bicycling, home or yard tasks, muscle-strengthening activities, and screen time (e.g., watching TV or using a computer). From 2007 to 2018, the classification of PA included two primary categories: work-related activities, which were divided into tasks of moderate and vigorous intensity, and leisure-time activities, which consisted of walking, cycling, as well as vigorous and moderate-intensity recreational activities (19). Cotinine, which is the primary byproduct of nicotine metabolism, was utilized to assess exposure to smoking by measuring cotinine concentrations in plasma samples (20). Alcohol intake was divided into three categories: individuals classified as heavy drinkers (with a daily intake of ≥15 g for women and ≥30 g for men), moderate drinkers (those consuming between 0 and 15 g per day for women and 0–30 g per day for men), and abstainers (<12 drinkers/year), scored as 2, 1, and 0, correspondingly (13). Detailed scoring criteria for each OBS component are provided in Supplementary Table Sl.

### 2.3 Evaluation of overweight/obesity

Overweight and obesity were assessed based on the BMI, which is calculated as weight (kg)/height (m)^2^. The CDC classifies BMI into specific categories: underweight (<18.5 kg/m^2^), normal weight (18.5–24.9 kg/m^2^), overweight (25–29.9 kg/m^2^), and obesity (≥30 kg/m^2^) (21).

### 2.4 Covariates

The analysis accounted for various covariates, including demographic and socioeconomic factors such as age, gender, and educational attainment (categorized as Less Than 9th Grade, 9–11th Grade, High School Graduate/GED or equivalent, Some College or Associate’s Degree, and College Graduate or higher). Ethnicity/race classifications included Mexican American, Other Hispanic, Non-Hispanic White, Non-Hispanic Black, and others. Marital status was grouped as married, single, or cohabiting. Economic status was assessed using the poverty income ratio, which was classified into low, medium, and high tiers. Smoking history referred to individuals who had smoked 100 cigarettes in their lives at least, while alcohol intake was defined as consuming a minimum of 12 beverages per annum. Medical history included prior diagnoses of hypertension and diabetes. In addition, total energy intake was included as a covariate in the analysis to account for dietary influences.

### 2.5 Ascertainment of mortality

This research examined two primary outcomes: mortality from all causes and mortality specifically attributed to cardiovascular disease (CVD). Information on mortality was obtained from the National Death Index (NDI) database. Cardiovascular disease (CVD) mortality was determined using diagnostic categories outlined in the 10th Revision of the International Classification of Diseases (ICD-10). The relevant codes include I00–I09, I11, I13, I20–I51, and I60–I69. NHANES baseline data (1999–2018) were matched longitudinally with the NDI database to access mortality information, with follow-up available until December 1, 2019. The NCHS provided the “*2019 Public-Use Linked Mortality Files*.”

### 2.6 Statistical analysis

All data analysis and graphical representation for this research were carried out utilizing R software (V4.4.1), IBM SPSS Statistics (V25), and GraphPad Prism (V9). Baseline characteristics were grouped according to OBS quartiles. The continuous variables were described using the mean and standard deviation (SD), while categorical variables were represented by frequencies and percentages. Differences in continuous variables between groups were examined using one-way analysis of variance (ANOVA), while Pearson’s chi-square test was employed for categorical variable analysis. Initially, the survival rates between different groups were compared using Kaplan–Meier (K–M) survival analysis, and the Log-rank test was employed to evaluate statistical significance. Subsequently, to evaluate the influence of various factors on the outcomes, Cox proportional hazard regression models were utilized, calculating hazard ratios (HRs) with 95% confidence intervals (CIs). To control for possible confounding variables, three distinct analytical models were constructed: Model 1 did not include any adjustments, while Model 2 controlled for age, race, and gender. In Model 3, further adjustments were made for age, gender, race, level of education, marital status, poverty-to-income ratio (PIR), alcohol use, smoking habits, as well as past medical history of hypertension and diabetes. To address potential non-linear relationships, Restricted Cubic Splines (RCS) were used to segment continuous variables, enabling a more refined characterization of their relationship with outcomes. Furthermore, subgroup analyses examine if the relationship between variables and outcomes varies across populations with differing characteristics. Subgroup analyses, along with sensitivity analysis, were conducted to verify the stability and generalizability of our results. In addition, we conducted a mediation analysis to investigate whether OBS could influence mortality risk in overweight/obese individuals through white blood cell count (WBC) and gamma-glutamyl transferase (GGT) (22, 23). We computed the average causal mediation effect (ACME), average direct effect (ADE), and proportion mediated through 500 simulations (sim). A *p*-value less than 0.05 indicates statistical significance.

## 3 Results

### 3.1 Baseline characteristics

Stratified by OBS quartiles, Table 1 displays the baseline characteristics. 26,219 overweight or obese participants were incorporated in the study. The participants’ average age in this study was 49.83 years, with a standard deviation of ±17.42 years, predominantly comprising non-Hispanic White individuals. Participants in the fourth quartile (Q4) of OBS were more inclined to be male, younger, married, and engage in alcohol consumption, while less likely to smoke or identify as non-Hispanic Black, in contrast to those in the first quartile (Q1). Those in the higher quartiles of OBS were more likely to possess higher educational qualifications, experience greater household earnings, and have a reduced rate of hypertension and diabetes.

**Table 1:** Baseline characteristics according to OBS quartiles in OV/OB patients.

	Q1	Q2	Q3	Q4	*p*-value
*N*	6,021	6,658	5,908	7,632	
Age, mean ± SD (years)	50.068 ± 18.013	50.934 ± 17.687	50.119 ± 17.233	48.458 ± 16.741	<0.001
Gender (%)					
Male	2014(33.4%)	2,799(42.0%)	3,110(52.6%)	5,112(67.0%)	<0.001
Female	4,007(66.6%)	3,859(58.0%)	2,798(47.4%)	2,520(33.0%)	
Race/ethnicity (%)					
Mexican American	1,065 (17.7%)	1,335 (20.1%)	1,203 (20.4%)	1,590 (20.8%)	<0.001
Other Hispanic	490 (8.1%)	619 (9.3%)	574 (9.7%)	631 (8.3%)	
Non-Hispanic White	2,297 (38.1%)	2,784 (41.8%)	2,679 (45.3%)	3,682 (48.2%)	
Non-Hispanic Black	1862 (30.9%)	1,540 (23.1%)	1,074 (18.2%)	1,165 (15.3%)	
Other Race	307 (5.1%)	380 (5.7%)	378 (6.4%)	564 (7.4%)	
Education (%)					
Less than 9th grade	821 (13.6%)	839 (12.6%)	673 (11.4%)	624 (8.2%)	<0.001
9–11th grade	1,117 (18.6%)	952 (14.3%)	757 (12.8%)	846 (11.1%)	
High school graduate/GED	1,580 (26.2%)	1,612 (24.2%)	1,305 (22.1%)	1,617 (21.2%)	
Some college or associate degree	1,677 (27.9%)	1994 (29.9%)	1774 (30.0%)	2,239 (29.3%)	
College graduate or above	705 (11.7%)	1,156 (17.4%)	1,320 (22.3%)	2,203 (28.9%)	
Not recorded	121 (2.0%)	105 (1.6%)	79 (1.3%)	103 (1.3%)	
PIR					
Low	2,473 (41.1%)	2076 (31.2%)	1,677 (28.4%)	1882 (24.7%)	<0.001
Middle	1784 (29.6%)	2,206 (33.1%)	1799 (30.5%)	2,229 (29.2%)	
High	1,258 (20.9%)	1830 (27.5%)	1944 (32.9%)	2,966 (38.9%)	
Not recorded	506 (8.4%)	546 (8.2%)	488 (8.3%)	555 (7.3%)	
Marital status					
Married	2,597 (43.1%)	3,473 (52.2%)	3,307 (56.0%)	4,586 (60.1%)	<0.001
Single	2,756 (45.8%)	2,585 (38.8%)	2063 (34.9%)	2,324 (30.5%)	
Living with a partner	477 (7.9%)	421 (6.3%)	417 (7.1%)	564 (7.4%)	
Not recorded	191 (3.2%)	179 (2.7%)	121 (2.0%)	158 (2.1%)	
Smoking					
Yes	2,883 (47.9%)	2,854 (42.9%)	2,556 (43.3%)	3,128 (41.0%)	<0.001
No	3,099 (51.5%)	3,774 (56.7%)	3,330 (56.4%)	4,470 (58.6%)	
Not recorded	39 (0.6%)	30 (0.5%)	22 (0.4%)	34 (0.4%)	
Drinking					
Yes	3,264 (54.2%)	3,737 (56.1%)	3,645 (61.7%)	4,921 (64.5%)	<0.001
No	2,756 (45.8%)	2,920 (43.9%)	2,260 (38.3%)	2,710 (35.5%)	
Not recorded	1 (0.0%)	1 (0.0%)	3 (0.1%)	1 (0.0%)	
Hypertension history					
Yes	2,550 (42.4%)	2,706 (40.6%)	2,298 (38.9%)	2,653 (34.8%)	<0.001
No	3,440 (57.1%)	3,926 (59.0%)	3,601 (61.0%)	4,965 (65.1%)	
Not recorded	31 (0.5%)	26 (0.4%)	9 (0.2%)	14 (0.2%)	
Diabetes status					
Diabetes	884 (14.7%)	972 (14.6%)	788 (13.3%)	856 (11.2%)	<0.001
No	4,975 (82.6%)	5,544 (83.3%)	4,968 (84.0%)	6,593 (86.4%)	
Prediabetes	157 (2.6%)	138 (2.1%)	147 (2.5%)	183 (2.4%)	
Not recorded	5 (0.1%)	4 (0.1%)	5 (0.1%)	0 (0.0%)	
OW/OB					
Overweight	2,489 (41.3%)	3,083 (46.3%)	2,877 (48.7%)	4,255 (55.8%)	<0.001
Obesity	3,532 (58.7%)	3,575 (53.7%)	3,031 (51.3%)	3,377 (44.2%)	

GED, general equivalent diploma; PIR, the ratio of family income to poverty. Continuous variables were presented as mean ± standard error, and categorical variables were reported as numbers (percentages).

### 3.2 The relationship between OBS and mortality

**Figure 2:**
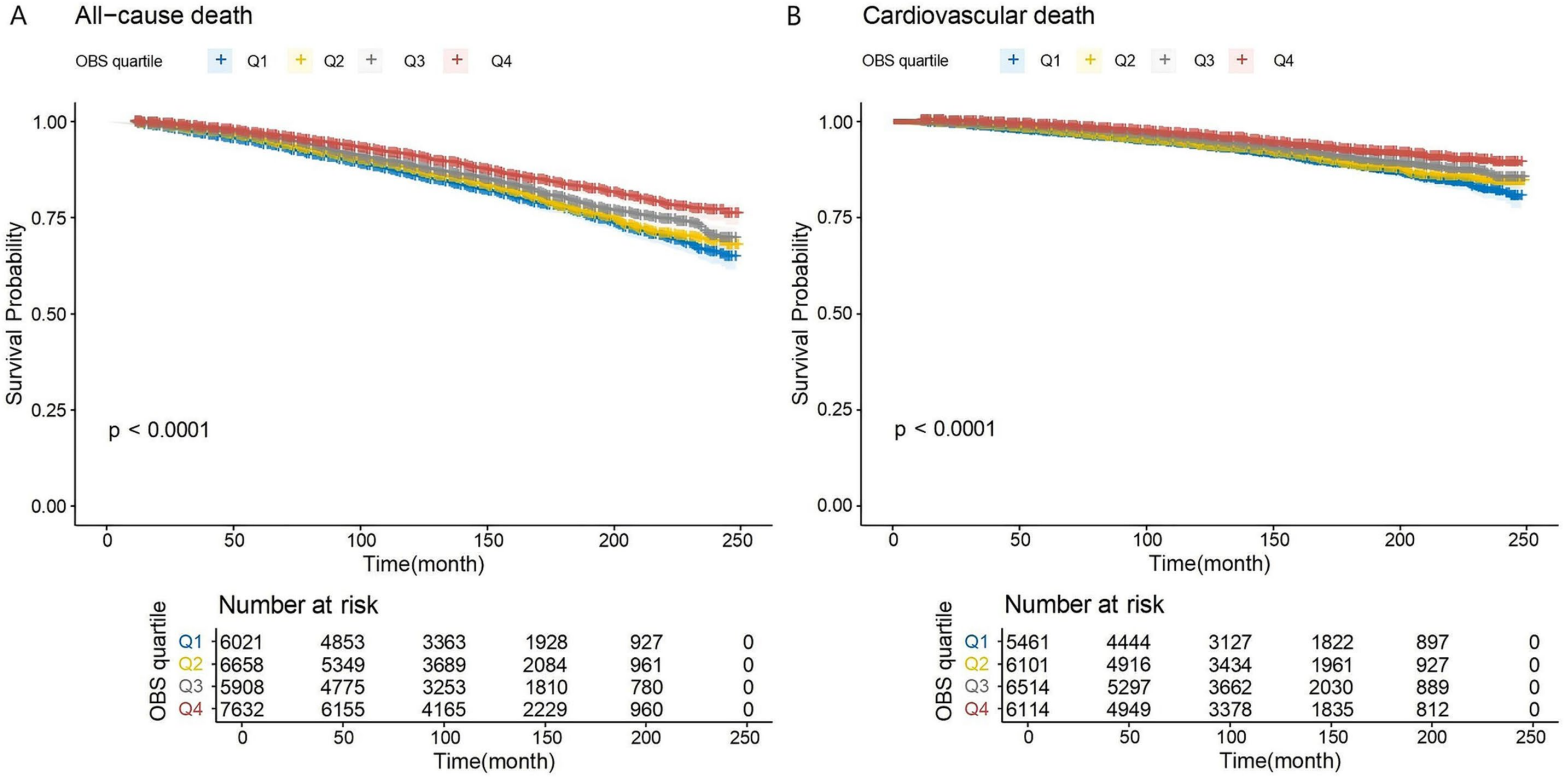
**(A,B)** K–M analyses for mortality among the four groups. Q1–Q4: quartiles 1–4. **(A)** All-cause mortality. **(B)** Cardiovascular mortality.

Figure 2 presents KM curves, illustrating statistical significance in all-cause and cardiovascular mortality across OBS levels (*p* < 0.001). Mortality was highest within the first OBS quartile (Q1) and lowest within the fourth quartile (Q4). With a follow-up duration averaging 115 months, among 26,219 overweight or obese participants, 2,239 (8.5%) deaths occurred, of which 837 (3.2%) were attributable to cardiovascular disease.

Table 2 shows the findings from the Cox regression. In the initial unadjusted model (Model 1), the risk of all-cause mortality reduced as OBS increased (HR 0.977, 95% CI 0.972–0.982, *p* < 0.001). When comparing the highest OBS quartile (Q4) to the lowest quartile (Q1), the former had a 35.0% lower risk of mortality (HR 0.650, 95% CI 0.591–0.714, *p* < 0.001). In Model 2, which controlled for age, sex, and race, individuals in the highest OBS quartile had a 35.4% reduction in mortality risk (HR 0.646, 95% CI 0.585–0.713, *p* < 0.001). In the fully adjusted model (Model 3), the risk of all-cause mortality was 21.7% lower for participants in Q4 compared to Q1 (HR 0.783, 95% CI 0.704–0.870, *p* < 0.001).

**Table 2:** Multivariable Cox regression models assessed the association between OBS and mortality in OV/OB.

Exposure	Model1	*P*	Model2	*P*	Model3	*P*
All-cause death						
OBS	0.977(0.972–0.982)	<0.001	0.975(0.970–0.980)	<0.001	0.985(0.980–0.991)	<0.001
OBS quartile						
Q1	Reference		Reference		Reference	
Q2	0.917(0.840–1.002)	0.055	0.824(0.754–0.900)	<0.001	0.916(0.833–1.007)	0.070
Q3	0.817(0.744–0.898)	<0.001	0.761(0.691–0.838)	<0.001	0.856(0.772–0.949)	0.003
Q4	0.650(0.591–0.714)	<0.001	0.646(0.585–0.713)	<0.001	0.783(0.704–0.870)	<0.001
Cardiovascular death						
OBS	0.973(0.966–0.980)	<0.001	0.971(0.963–0.979)	<0.001	0.983(0.974–0.992)	<0.001
OBS quartile						
Q1	Reference		Reference		Reference	
Q2	0.918(0.803–1.050)	0.212	0.816(0.713–0.934)	0.003	0.920(0.796–1.062)	0.255
Q3	0.763(0.665–0.877)	<0.001	0.728(0.632–0.840)	<0.001	0.815(0.700–0.948)	0.008
Q4	0.568(0.486–0.663)	<0.001	0.570(0.484–0.670)	<0.001	0.705(0.592–0.839)	<0.001

Model 1 was unadjusted; Model 2 adjusted for age, race, and sex; and Model 3 further adjusted for age, sex, race, education level, marital status, poverty-to-income ratio, alcohol consumption, smoking status, history of hypertension, and history of diabetes.

The Cox regression analysis of OBS and cardiovascular mortality is shown in Table 2. In the unadjusted model (Model 1), the risk of cardiovascular mortality reduced as OBS increased (HR 0.973, 95% CI 0.966–0.980, *p* < 0.001). Participants in Q4 demonstrated a 43.2% lower cardiovascular mortality risk compared to those in Q1 (HR 0.568, 95% CI 0.486–0.663, *p* < 0.001). In Model 2, individuals in the Q4 group still showed a 43.0% reduction in cardiovascular mortality risk (HR 0.570, 95% CI 0.484–0.670, *p* < 0.001). In the fully adjusted Model 3, an inverse relationship between OBS and cardiovascular mortality persisted (HR 0.983, 95% CI 0.974–0.992, *p* < 0.001). Participants categorized in Q4 exhibited a 29.5% lower risk of cardiovascular mortality compared to those in Q1 (HR 0.705, 95% CI 0.592–0.839, *p* < 0.001).

Based on model 3, we added total energy intake as a covariate for sensitivity analysis (Supplementary Table S2). The results indicate that the association between OBS and both all-cause mortality and cardiovascular mortality remains statistically significant. In Supplementary Table S3, we provide a more detailed breakdown of the differential contributions of OBS categories to mortality outcomes. In the fully adjusted model, both dietary OBS (HR 0.989, 95% CI 0.983–0.994, *p* < 0.001) and lifestyle OBS (HR 0.911, 95% CI 0.887–0.936, *p* < 0.001) were negatively associated with all-cause mortality, and the results were statistically significant. After full adjustment, the risk of cardiovascular mortality decreased with the increase in both dietary OBS (HR 0.983, 95% CI 0.974–0.992, *p* < 0.001) and lifestyle OBS (HR 0.898, 95% CI 0.860–0.938, *p* < 0.001).

**Figure 3:**
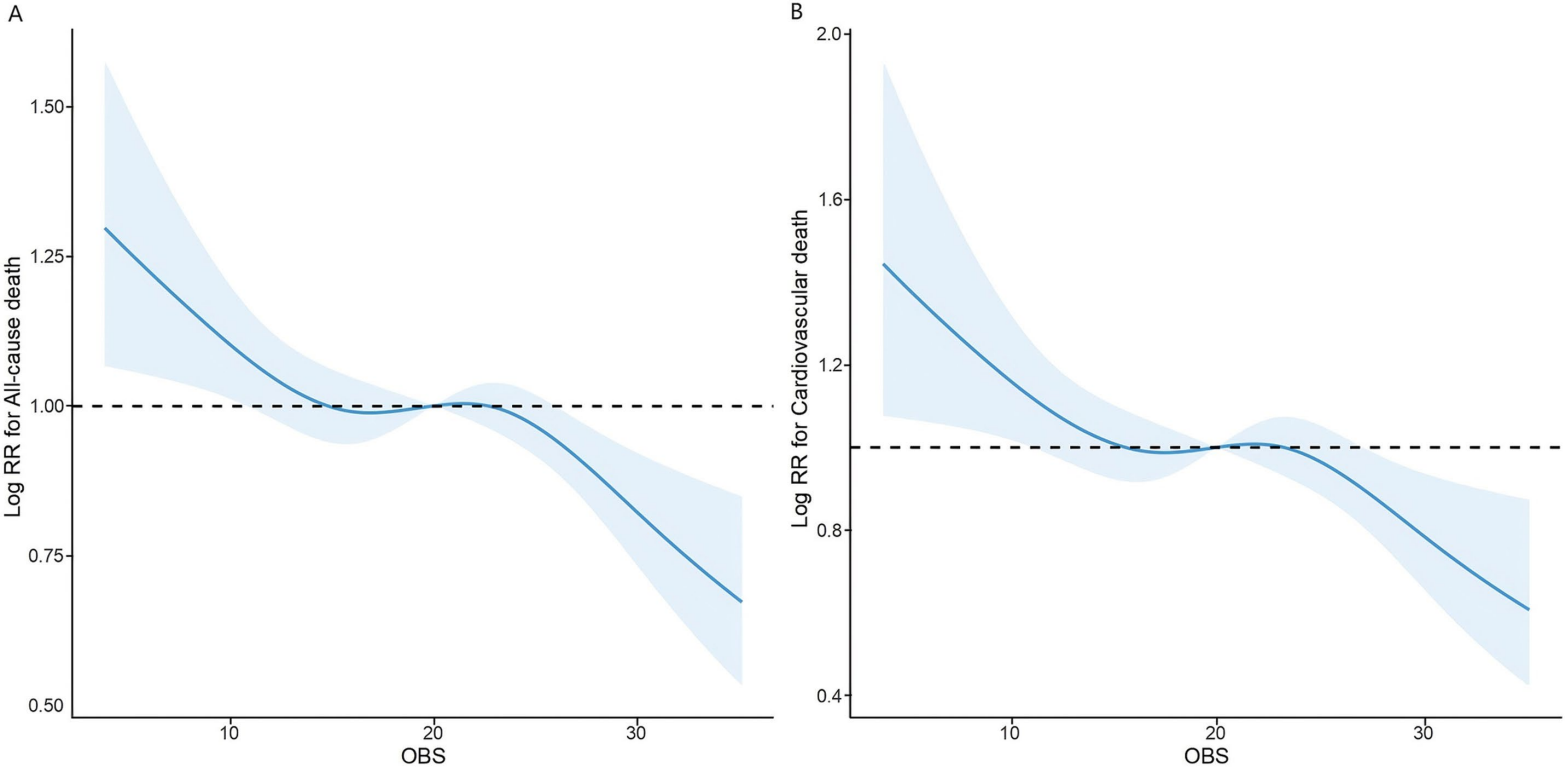
The association of OBS with all-cause **(A)** and cardiovascular mortality **(B)** among overweight and obese visualized by restricted cubic spline.

We further optimized the RCS analysis based on the completely adjusted model. The findings suggested a linear inverse relationship between OBS and both all-cause and cardiovascular mortality, with non-linearity *p*-values of 0.1816 and 0.0798, respectively (Figure 3).

### 3.3 Subgroup analysis

To evaluate the robustness of the relationship between OBS and mortality, the dataset was divided into subcategories according to variables such as age, gender, ethnicity, educational attainment, marital status, income level, smoking and drinking behaviors, as well as histories of hypertension, diabetes, and overweight/obesity (Table 3). The findings demonstrated that the inverse relationship between OBS and mortality remained broadly uniform across various subgroups. Our analysis revealed that the impact of OBS on all-cause mortality was significantly altered by overweight/obesity within this population subgroup (*p* for interaction = 0.002). Age, level of education, and overweight/obesity were recognized as significant modifiers affecting the link between OBS and cardiovascular mortality among overweight or obese individuals (*p* for interaction = 0.039, 0.014, and 0.017, correspondingly). In addition, although the interaction *p*-value for the diabetes subgroup analysis was not statistically significant, the observed risk differences suggest a potential trend. Specifically, individuals with diabetes (HR 0.993, 95% CI 0.982–1.004, *p* = 0.205) seem to derive less benefit from higher OBS compared to those with normal glucose metabolism (HR 0.984, 95% CI 0.978–0.990, *p* < 0.001). Therefore, we conducted a subgroup analysis between the impaired glucose metabolism group (pre-diabetes + diabetes group) and the non-diabetic group. The results suggest that impaired glucose metabolism is an important factor influencing the association between OBS and cardiovascular mortality in overweight or obese individuals (*p* for interaction < 0.001) (Supplementary Table S4).

**Table 3:** Stratified analysis of the relationship between OBS and mortality in overweight and obesity.

	All-cause mortality		Cardiovascular mortality	
	HR (95% CI)	*P*-value	HR (95% CI)	*P*-value
Age				
≤60	0.987(0.977–0.998)	0.022	0.979(0.960–0.998)	0.029
>60	0.986(0.980–0.992)	<0.001	0.984(0.975–0.993)	0.001
*P* for interaction		0.080		0.039
Gender				
Male	0.988(0.981–0.996)	0.002	0.988(0.977–0.999)	0.031
Female	0.984(0.976–0.993)	<0.001	0.977(0.964–0.990)	0.001
*P* for interaction		0.550		0.182
Race				
Mexican American	0.991(0.976–1.006)	0.222	0.989(0.966–1.013)	0.359
Other Hispanic	1.002(0.974–1.031)	0.894	1.006(0.962–1.052)	0.787
Non-Hispanic White	0.984(0.977–0.991)	<0.001	0.982(0.971–0.992)	0.001
Non-Hispanic Black	0.986(0.973–0.998)	0.027	0.977(0.958–0.996)	0.016
Other Race	0.989(0.955–1.025)	0.549	1.027(0.965–1.094)	0.401
*P* for interaction		0.257		0.361
Education				
Less than 9th grade	0.995(0.981–1.008)	0.421	0.992(0.974–1.011)	0.431
9–11th grade	0.986(0.974–0.999)	0.035	0.988(0.970–1.008)	0.238
High school graduate/GED	0.993(0.982–1.003)	0.183	0.997(0.980–1.013)	0.696
Some college or associate degree	0.981(0.970–0.992)	0.001	0.968(0.950–0.985)	<0.001
College graduate or above	0.976(0.962–0.990)	0.001	0.966(0.944–0.988)	0.002
*P* for interaction		0.055		0.014
Marital status				
Married	0.987(0.979–0.994)	0.001	0.988(0.977–1.000)	0.048
Single	0.986(0.978–0.994)	0.001	0.979(0.966–0.991)	0.001
Living with a partner	0.992(0.961–1.024)	0.629	0.985(0.926–1.047)	0.630
*P* for interaction		0.967		0.689
Poverty to income ratio				
Low	0.990(0.981–0.998)	0.018	0.983(0.970–0.997)	0.013
Middle	0.986(0.977–0.995)	0.002	0.988(0.975–1.002)	0.083
High	0.982(0.971–0.993)	0.001	0.975(0.957–0.993)	0.007
*P* for interaction		0.166		0.143
Smoking				
Yes	0.988(0.981–0.995)	0.001	0.986(0.975–0.998)	0.017
No	0.985(0.977–0.994)	0.001	0.981(0.969–0.994)	0.003
*P* for interaction		0.870		0.301
Drinking				
Yes	0.985(0.978–0.992)	<0.001	0.977(0.966–0.988)	<0.001
No	0.988(0.980–0.997)	0.005	0.992(0.979–1.004)	0.178
*P* for interaction		0.560		0.135
Hypertension history				
Yes	0.984(0.977–0.991)	<0.001	0.579(0.497–0.675)	<0.001
No	0.990(0.982–0.999)	0.030	0.456(0.366–0.568)	<0.001
*P* for interaction		0.843		0.900
Diabetes status				
Diabetes	0.993(0.982–1.004)	0.205	0.988(0.972–1.005)	0.172
No	0.984(0.978–0.990)	<0.001	0.982(0.972–0.992)	<0.001
Prediabetes	0.990(0.960–1.022)	0.535	0.975(0.929–1.024)	0.308
*P* for interaction		0.411		0.801
OW/OB				
Overweight	0.981(0.974–0.989)	<0.001	0.535(0.449–0.639)	<0.001
Obesity	0.990(0.982–0.998)	0.014	0.538(0.450–0.644)	<0.001
*P* for interaction		0.002		0.017

The stratified analysis was performed using the fully adjusted model (Model 3).

### 3.4 Mediation analyses

**Figure 4:**
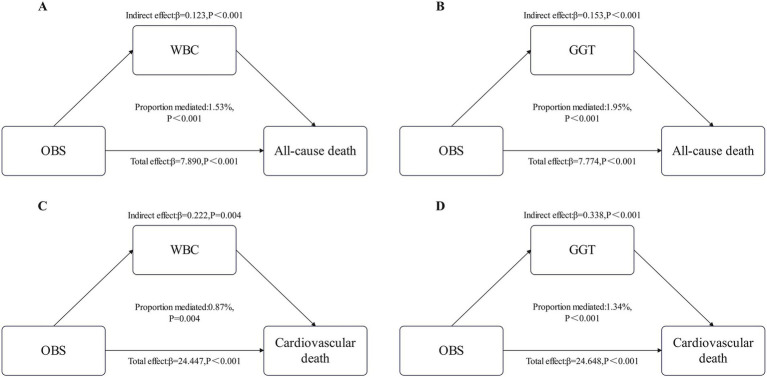
Estimated proportion of the association between OBS and All-cause death mediated by WBC **(A)** and GGT **(B)**. Estimated proportion of the association between OBS and Cardiovascular death mediated by WBC **(C)** and GGT **(D)**. WBC, white blood cell; GGT, gamma-glutamyl transferase.

The WBC and GGT mediated the relationship between OBS and all-cause mortality, with mediation proportions of 1.53 and 1.95% (*p* < 0.001), respectively. Similarly, WBC and GGT also mediated the relationship between OBS and cardiovascular mortality, with mediation proportions of 0.87 and 1.34% (*p* < 0.05), respectively (Figure 4).

## 4 Discussion

As far as we are aware, this investigation represents the first attempt to explore the relationship between OBS and survival outcomes among overweight and obese individuals, utilizing data derived from the NHANES database. In a cohort of 26,219 overweight or obese individuals throughout 10 NHANES cycles (1999–2018), we found an inverse association between OBS and the risks associated with all-cause as well as cardiovascular mortality, with consistent and stable results across subgroup analyses. These findings suggest that antioxidant-rich diets and lifestyle patterns may confer a protective effect on the survival outcomes of overweight and obese individuals.

Reactive oxygen species are by-products of metabolic pathways in the body, strongly associated with the advancement of overweight, obesity, and related metabolic complications. The generation of ROS predominantly occurs in the mitochondria within cells (24). Overweight and obese increase the mechanical load on the body and myocardial metabolism, thereby leading to increased oxygen consumption. Additionally, inflammatory mediators and bioactive substances, such as TNF-*α*, interleukins (IL), and angiotensin II, are secreted by adipose tissue and play a pivotal role in stimulating immune cells and activating NADPH oxidase (NOS), which consequently leads to the generation of ROS (25–27). Similarly, ROS production promotes the secretion of inflammatory factors and pro-inflammatory transcription mediators, such as nuclear factor activator protein-1 (AP-1) and kappa B (NF-κB) (28). Furthermore, ROS plays a critical role in adipogenesis by triggering the differentiation of mesenchymal stem/stromal cells into adipocytes and activating adipogenic signaling networks via second messengers (25, 29). Increased ROS in tissues leads to the inactivation of components and enzymes in the respiratory chain, resulting in mitochondrial dysfunction. These processes ultimately contribute to multiple metabolic diseases, such as obesity, type 2 diabetes, aging, and may even increase mortality (30).

Several large-scale cohort studies have shown that the risk of mortality is elevated in overweight and obese populations (31). Our results demonstrate that increased OBS levels are linked to a lower likelihood of both all-cause and cardiovascular mortality in individuals with overweight or obesity, consistent with previous research. Cheng et al. (32) conducted an analysis using seven NHANES cycles and discovered that the link between OBS and the risk of cardiovascular disease was more pronounced among individuals exhibiting greater metabolic abnormalities. Wang et al. (33), utilizing a cross-sectional approach, identified an inverse relationship between OBS and both abdominal adiposity and visceral fat mass. High-quality diets may reduce the risks of cardiovascular and all-cause mortality associated with inflammation, with this effect being particularly pronounced in obese populations (34). High-quality diets are typically distinguished by the intake of abundant fruits, nuts, legumes, and vegetables, which are abundant in antioxidants, unsaturated fatty acids, various vitamins, and dietary fiber (35, 36). Obese individuals with mild to moderate inflammation can significantly reduce their all-cause mortality risk by strictly adhering to a healthy diet, though this effect is not significant in those with severe inflammation. This suggests that early intervention in systemic inflammation in overweight and obese individuals could reduce mortality risk (37). Nevertheless, several studies have reported a lack of a significant association between specific dietary factors and the risk of mortality (38). This discrepancy may stem from interactions between dietary nutrients or the impact of food production and processing stages (39, 40). While there are limited studies specifically examining the effect of OBS on mortality in overweight and obese individuals, various prospective cohort researches have analyzed the association between individual or select OBS components, such as iron, vitamin C, and *β*-carotene, and mortality. These studies indicate that elevated levels of antioxidants are linked to a lower incidence of mortality (41, 42). A cohort study including 29,836 obese adults found that moderate to intense exercise effectively reduced the probability of microvascular issues, heart-related diseases, and death from any cause in obese individuals (43). Lai et al. (22), after analyzing 10 cycles of NHANES data, found that WBC and GGT played a potential mediating role between OBS and mortality risk in individuals with cardiometabolic risk factors. OBS provides a comprehensive and objective assessment of antioxidant-rich diets and lifestyles. Previous studies have analyzed the association among OBS and mortality risk, with findings suggesting an inverse association between OBS and mortality rates (17, 38, 44, 45). Romacho et al. (46) carried out a non-randomized controlled study, finding that lifestyle interventions improved systemic inflammation and reduced cardiovascular disease risk in obese patients. Kc et al. (47) discovered that educational level was linked to mortality risk, and we identified a potential interaction between education level and OBS concerning cardiovascular mortality risk in overweight/obese populations.

This study possesses several strengths. First, Multi-stage probability sampling design was utilized in the NHANES data collection to ensure that the sample and data are nationally representative. Second, OBS is a composite index that, compared to individual indicators, provides a more comprehensive analysis of the correlation between oxidative stress and mortality in overweight and obese populations. Thirdly, the data spanning from 1999 to 2018, with a large sample size and extended follow-up period, enhances the credibility as well as the stability of the outcomes. Finally, our study controlled for several potential confounding factors, including demographic characteristics, history of hypertension, smoking, and alcohol consumption.

However, there are several limitations to this study. First, although OBS includes 20 factors, it does not encompass all oxidative stress-related factors, leaving room for further adjustment. Second, dietary data were based solely on the first dietary interview, and may not accurately reflect participants’ typical diets, potentially introducing bias into the results. Third, self-reported data from participants may be subject to reporting bias, particularly regarding dietary and lifestyle factors. Fourth, the findings may not be fully applicable to non-U.S. populations or individuals outside the overweight and obese categories. Therefore, caution should be exercised when applying these results to non-U.S. populations or individuals within the normal weight range. Further research in more diverse and broader populations is needed in the future. Fifth, a potential limitation of this study is the reliance on National Death Index (NDI) data to ascertain mortality outcomes. Due to inherent limitations in coding practices and classification systems, misclassification of causes of death may still occur. Finally, while this study utilized large-scale longitudinal data from the NHANES, there is an inherent risk of selection bias due to incomplete data. Some participants were excluded from the analysis because of missing data or other reasons, and as a result, selection bias to some extent is unavoidable.

## 5 Conclusion

Our study, encompassing 26,219 participants spanning 10 NHANES cycles, demonstrates a negative association between OBS and both all-cause and cardiovascular mortality in overweight and obese populations. A higher OBS score indicates higher exposure to antioxidant factors in diet and lifestyle and correlates with a reduced risk of both all-cause and cardiovascular mortality. The study emphasizes the status of maintaining an antioxidant-enriched diet, along with a healthy lifestyle, as part of management strategies for overweight and obesity.

## Data Availability

The datasets presented in this study can be found in online repositories. The names of the repository/repositories and accession number(s) can be found in the article/Supplementary material.
