# Supplementary material for: Association of oxidative balance score with all-cause and cardiovascular mortality in overweight and obese
Source: Front Nutr. 2025 Jan 28;12:1536024. doi: 10.3389/fnut.2025.1536024 (PMC11810733; doi:10.3389/fnut.2025.1536024)
Supplement: Supplementary file 1 [file Table_1.DOCX]

Supplementary Table l: Oxidative balance score allocation scheme.

|  |  |  | **Male** |  |  | **Female** |  |
| --- | --- | --- | --- | --- | --- | --- | --- |
| **OBS components** | Property | 0 | 1 | 2 | 0 | 1 | 2 |
| **Dietary OBS components** |  |  |  |  |  |  |  |
| Dietary fiber (g/d) | A | <12.10 | 12.10-20.30 | ≥20.30 | <10.00 | 10.00-16.50 | ≥16.50 |
| Carotene (RE/d) | A | <96.54 | 96.54-312.34 | ≥312.34 | <96.23 | 96.23-376.47 | ≥376.47 |
| Riboflavin (mg/d) | A | <1.66 | 1.66-2.59 | ≥2.59 | <1.28 | 1.28-1.97 | ≥1.97 |
| Niacin (mg/d) | A | <20.85 | 20.85-32.04 | ≥32.04 | <14.52 | 14.52-22.45 | ≥22.45 |
| Vitamin B_6_ (mg/d) | A | <1.59 | 1.59-2.51 | ≥2.51 | <1.13 | 1.13-1.81 | ≥1.81 |
| Total folate (mcg/d) | A | <308 | 308-496 | ≥496 | <237 | 237-378 | ≥378 |
| Vitamin B_12_ (mcg/d) | A | <3.16 | 3.16-6.14 | ≥6.14 | <2.15 | 2.15-4.29 | ≥4.29 |
| Vitamin C (mg/d) | A | <40.05 | 32.20-100.10 | ≥95.10 | <29.9 | 29.9-88.8 | ≥88.8 |
| Vitamin E (ATE) (mg/d) | A | <5.42 | 5.42- 9.48 | ≥9.48 | <4.44 | 4.44-7.61 | ≥7.61 |
| Calcium (mg/d) | A | < 645.00 | 645.00-1103.52 | ≥1103.52 | <534 | 534-899 | ≥899 |
| Magnesium (mg/d) | A | <245.00 | 245.00-365.93 | ≥365.93 | <191 | 191-282 | ≥282 |
| Zinc (mg/d) | A | <9.14 | 9.14-14.62 | ≥14.62 | <6.57 | 6.57-10.34 | ≥10.34 |
| Copper (mg/d) | A | <1.01 | 1.01-1.52 | ≥1.52 | <0.80 | 0.80-1.20 | ≥1.20 |
| Selenium (mcg/d) | A | <94.30 | 94.30-144.70 | ≥144.70 | <67.90 | 67.90-103.98 | ≥103.98 |
| Total fat (g/d) | P | ≥65.66 | 65.66-104.56 | <104.56 | ≥48.89 | 48.89-77.58 | <77.58 |
| Iron (mg/d) | P | ≥11.93 | 11.93-18.59 | <18.59 | ≥9.01 | 9.01-13.90 | <13.90 |
| **Lifestyle OBS components** |  |  |  |  |  |  |  |
| Physical activity (MET-h/week) | A | <16.00 | 16.00-50.32 | ≥50.32 | <6.67 | 6.67-32.55 | ≥32.55 |
| Alcohol (g/d) | P | ≥30 | 0-30 | None | ≥15 | 0-15 | None |
| Body mass index (kg/m^2^) | P | ≥25.57 | 25.57-29.80 | <29.80 | ≥25.21 | 25.21-31.30 | <31.30 |
| Cotinine (ng/mL) | P | ≥0.029 | 0.029-0.839 | <0.839 | ≥0.019 | 0.019-0.126 | <0.126 |

OBS: oxidative balance score; A: antioxidant; P: prooxidant; RE: retinol equivalent; ATE: alpha-tocopherol equivalent; MET: metabolic equivalent.

Supplementary Table 2: Sensitivity analysis on the association between OBS and mortality by adding total energy intake in model 3.

| **Exposure** | **All-cause death** | | **Cardiovascular death** | |
| --- | --- | --- | --- | --- |
|  | Model 3+ total energy intake | P | Model 3+ total energy intake | P |
| **OBS quartile** |  |  |  |  |
| Q1 | Reference |  | Reference |  |
| Q2 | 0.884(0.801-0.976) | 0.015 | 0.947(0.813-1.103) | 0.485 |
| Q3 | 0.800(0.714-0.896) | <0.001 | 0.842(0.706-1.004) | 0.055 |
| Q4 | 0.701(0.615-0.800) | <0.001 | 0.618(0.489-0.781) | <0.001 |
| **OBS** | 0.977(0.970-0.984) | <0.001 | 0.969(0.958-0.980) | <0.001 |

Model 3 adjusted for age, sex, race, education level, marital status, poverty-to-income ratio, alcohol consumption, smoking status, history of hypertension, and history of diabetes. Total energy intake, derived from dietary data, refers to the total energy consumed over a 24-hour period.

Supplementary Table 3: Associations between OBS and the risk of mortality in different OBS categories.

|  | **Model 1** | | **Model 2** | | **Model 3** | |
| --- | --- | --- | --- | --- | --- | --- |
|  | HR(95%CI) | P value | HR(95%CI) | P value | HR(95%CI) | P value |
| **All-cause death** |  |  |  |  |  |  |
| Dietary OBS | 0.976(0.971-0.980) | ＜0.001 | 0.980(0.975-0.985) | ＜0.001 | 0.989(0.983-0.994) | ＜0.001 |
| Lifestyle OBS | 1.008(0.985-1.030) | 0.499 | 0.875(0.855-0.896) | ＜0.001 | 0.911(0.887-0.936) | ＜0.001 |
| **Cardiovascular death** |  |  |  |  |  |  |
| Dietary OBS | 0.968(0.961-0.976) | ＜0.001 | 0.974(0.965-0.982) | ＜0.001 | 0.983(0.974-0.992) | ＜0.001 |
| Lifestyle OBS | 1.024(0.988-1.062) | 0.186 | 0.862(0.830-0.896) | ＜0.001 | 0.898(0.860-0.938) | ＜0.001 |

Dietary OBS: dietary fiber, carotene, riboflavin, niacin, vitamin B6, total folate, vitamin B12, vitamin C, vitamin E, calcium, magnesium, zinc, copper, selenium, total fat, and iron. Lifestyle OBS: physical activity, body mass index (BMI), alcohol consumption, and smoking.

Supplementary Table 4: Stratified analysis of the association between OBS and mortality in overweight and obese individuals with respect to glucose metabolism.

|  | **All-cause mortality** | | **Cardiovascular mortality** | |
| --- | --- | --- | --- | --- |
|  | HR (95% CI) | P value | HR (95% CI) | P value |
| **Diabetes status** |  |  |  |  |
| No | 0.985(0.979-0.991) | ＜0.001 | 0.976(0.966-0.986) | ＜0.001 |
| DM+ preDM | 0.991(0.977-1.005) | 0.223 | 0.987(0.971-1.004) | 0.122 |
| P for interaction |  | 0.536 |  | ＜0.001 |

DM: Diabetes Mellitus; preDM: pre-diabetes mellitus, a condition where blood sugar levels are higher than normal but not yet high enough to be diagnosed as type 2 diabetes.
